# Health effects and wind turbines: A review of the literature

**DOI:** 10.1186/1476-069X-10-78

**Published:** 2011-09-14

**Authors:** Loren D Knopper, Christopher A Ollson

**Affiliations:** 1 Intrinsik Environmental Sciences Inc., 1790 Courtwood Crescent, Ottawa ON, K2C 2B5, Canada; 2 Intrinsik Environmental Sciences Inc., 6605 Hurontario Street, Suite 500, Mississauga, ON, L5T 0A3, Canada

**Keywords:** Wind turbines, health, annoyance, infrasound, sound pressure level, noise

## Abstract

**Background:**

Wind power has been harnessed as a source of power around the world. Debate is ongoing with respect to the relationship between reported health effects and wind turbines, specifically in terms of audible and inaudible noise. As a result, minimum setback distances have been established world-wide to reduce or avoid potential complaints from, or potential effects to, people living in proximity to wind turbines. People interested in this debate turn to two sources of information to make informed decisions: scientific peer-reviewed studies published in scientific journals and the popular literature and internet.

**Methods:**

The purpose of this paper is to review the peer-reviewed scientific literature, government agency reports, and the most prominent information found in the popular literature. Combinations of key words were entered into the Thomson Reuters Web of Knowledge^SM ^and the internet search engine Google. The review was conducted in the spirit of the evaluation process outlined in the Cochrane Handbook for Systematic Reviews of Interventions.

**Results:**

Conclusions of the peer reviewed literature differ in some ways from those in the popular literature. In peer reviewed studies, wind turbine annoyance has been statistically associated with wind turbine noise, but found to be more strongly related to visual impact, attitude to wind turbines and sensitivity to noise. To date, no peer reviewed articles demonstrate a direct causal link between people living in proximity to modern wind turbines, the noise they emit and resulting physiological health effects. If anything, reported health effects are likely attributed to a number of environmental stressors that result in an annoyed/stressed state in a segment of the population. In the popular literature, self-reported health outcomes are related to distance from turbines and the claim is made that infrasound is the causative factor for the reported effects, even though sound pressure levels are not measured.

**Conclusions:**

What both types of studies have in common is the conclusion that wind turbines can be a source of annoyance for some people. The difference between both types is the reason for annoyance. While it is acknowledged that noise from wind turbines can be annoying to some and associated with some reported health effects (e.g., sleep disturbance), especially when found at sound pressure levels greater than 40 db(A), given that annoyance appears to be more strongly related to visual cues and attitude than to noise itself, self reported health effects of people living near wind turbines are more likely attributed to physical manifestation from an annoyed state than from wind turbines themselves. In other words, it appears that it is the change in the environment that is associated with reported health effects and not a turbine-specific variable like audible noise or infrasound. Regardless of its cause, a certain level of annoyance in a population can be expected (as with any number of projects that change the local environment) and the acceptable level is a policy decision to be made by elected officials and their government representatives where the benefits of wind power are weighted against their cons. Assessing the effects of wind turbines on human health is an emerging field and conducting further research into the effects of wind turbines (and environmental changes) on human health, emotional and physical, is warranted.

## Background

Wind power has been identified as a clean renewable energy source that does not contribute to global warming and is without known emissions or harmful wastes [1]. Studies on public attitudes in Europe and Canada show strong support for the implementation of wind power [2]. Indeed, wind power has become an integrated part of provincial energy strategies across Canada; in Ontario, the Ontario Power Authority has placed a great deal of emphasis on procuring what they term "renewable and cleaner sources of electricity", such as wind [3].

Although wind power has been harnessed as a source of electricity for several decades around the world, its widespread use as a significant source of energy in Ontario is relatively recent. As with the introduction of any new technology, concerns have been raised that wind power projects could lead to impacts on human health. These concerns are related to two primary issues: wind turbine design and infrastructure (i.e., electromagnetic frequencies from transmission lines, shadow flicker from rotor blades, ice throw from rotor blades and structural failure) and wind turbine noise (i.e., levels of audible noise [including low frequency noise] and infrasound). If left unchecked and unmanaged, it is possible that individually or cumulatively, these issues could lead to potential health impacts. In terms of noise, high sound pressure levels (loudness) of audible noise and infrasound have been associated with learning, sleep and cognitive disruptions as well as stress and anxiety [4-8].

As a result, minimum setback distances have been established world-wide to reduce or avoid potential effects for people living in proximity to wind turbines. Under the Ontario Renewable Energy Approval (REA) Regulation (O. Reg. 359/09, as amended by O. Reg. 521/10), a minimum setback distance of 550 m must exist between the centre of the base of the wind turbine and the nearest noise receptor (e.g., a building or campground). This minimum setback distance was developed through noise modeling under worst-case conditions to give a conservative estimate of the required distance to attain a sound level of 40 dB(A) [9], the noise level that corresponds to the WHO (Europe) night-noise guideline, a health-based limit value "necessary to protect the public, including most of the vulnerable groups such as children, the chronically ill and the elderly, from the adverse health effects of night noise" [8]. Globally, rural residential noise limits are generally set at 35 to 55 dB(A) [10].

This paper focuses on the research involving land-based wind turbine projects. There are several international off-shore marine projects that are in operation. There was considerable interest in Ontario in developing off-shore wind projects on the Great Lakes. However, in February, 2011 the Province announced that it would not proceed with proposed offshore wind projects until further scientific research is conducted http://www.news.ontario.ca/ene/en/2011/02/ontario-rules-out-offshore-wind-projects.html. This does not appear to have been related, however, to health concerns.

Regardless, debate is ongoing with respect to the relationship between reported health effects and wind turbines, specifically in terms of audible and inaudible noise. People interested in this debate tend to turn to two sources of information in order to make decisions: scientific peer-reviewed studies published in scientific journals, and the popular literature and internet. For the general public, the latter sources are the most readily available and numerous websites have been constructed by individuals or groups to support or oppose the development of wind farms. Often these websites state the perceived impacts on, or benefits to, human health to support the position of the individual or group. The majority of information posted on these websites cannot be traced back to a scientific peer-reviewed source and is typically anecdotal in nature. This serves to spread misconceptions about the potential impacts of wind energy on human health making it difficult for the general public (and scientists) to ascertain which claims can be substantiated by scientific evidence.

Accordingly, the purpose of this paper is to provide results of a review of the peer-reviewed scientific literature and the most prominent information found in the popular literature. We have selected this journal as the source of publication because it is a scientifically credible journal with peer-reviewed articles that are easily accessible by the general population who are interested in the subject of wind turbines and health effects. Results of this review are used to draw conclusions about wind turbines and health effects using a weight-of-evidence approach.

## Methods

### Peer-Reviewed Literature

Publication of scientific findings is the basis of scientific discourse, communication and debate. The peer review process is considered a fundamental tenet of quality control in scientific publishing. Once a research paper has been submitted to a journal for publication it is reviewed by external independent experts in the field. The experts review the validity, reliability and importance of the results and recommend that the manuscript be accepted, revised or rejected. This process, though not perfect, ensures that the methods employed and the findings of the research receive a high level of scrutiny, such that an independent researcher could repeat the experiment or calculation of results, prior to their publication. This process seeks to ensure that the published research is of a high standard of quality, accurate, can be reproduced and demonstrates academic/professional integrity.

In order to assess peer-reviewed studies designed to test hypotheses about the association between potential health effects in humans and wind turbines, a review of the primary scientific literature was conducted. While our review did not strictly follow the evaluation process outlined in the Cochrane Handbook for Systematic Reviews of Interventions [11], the standard for conducting information reviews in healthcare and pharmaceutical industries, it was conducted in the spirit of the Cochrane systematic review in that it was designed based on the principle that "science is cumulative", and by considering all available evidence, decisions could be made that reflect the best science available. It also involves critical review and critique of the published literature and at times weighting some manuscripts over others in the same scientific field.

To facilitate this review, combinations of key words (i.e., annoyance, noise, environmental change, sleep disturbance, epilepsy, stress, health effect(s), wind farm(s), infrasound, wind turbines(s), low frequency noise, wind turbine syndrome, neighborhood change) were selected and entered into the Thomson Reuters (formerly ISI) Web of Knowledge^SM^. The Web of Knowledge^SM ^is a database that covers over 10,000 high-impact journals in the sciences, social sciences, and arts and humanities, as well as international proceedings coverage for over 120,000 conferences. The Web of Knowledge^SM ^comprises seven citation databases, two of which are relevant to the search: the Science Citation Index Expanded (SCI-Expanded) and the Social Sciences Citation Index (SSCI). The SCI-Expanded includes over 6,650 major journals across 150 scientific disciplines and includes all cited references captured from indexed articles. Coverage of the literature spans the year 1900 to the present. On average, 19,000 new records per week are added to the SCI-Expanded. SSCI is a multidisciplinary index of the social sciences literature. SSCI includes over 1,950 journals across 50 social sciences disciplines from the year 1956 to the present. It averages 2,900 new records per week. Use of this literature search platform means the most up-to-date multidisciplinary studies published and peer-reviewed could be obtained.

Although hundreds of articles were found during the search, very few were related to the association between potential health effects and wind turbines. For example, numerous articles have been published about infrasound, but very few have been published about infrasound and wind turbines. Indeed, only fifteen articles, published between 2003 and 2011, were found relevant [12-26]. What can be seen from these articles is that the relationship between wind turbines and human responses to them is extremely complex and influenced by numerous variables, the majority of which are nonphysical. What is clear is that some people living near wind turbines experience annoyance due to wind turbines, and visual impact tends to be a stronger predictor of noise annoyance than wind turbine noise itself. Swishing, whistling, resounding and pulsating/throbbing are sound characteristics most highly correlated with annoyance by wind turbine noise for those people who noticed the noise outside their dwellings. Some people are also disturbed in their sleep by wind turbines. In general, five key points have come out of these peer-reviewed studies with regards to health and wind turbines.

### 1. People tend to notice sound from wind turbines almost linearly with increasing sound pressure level

In the studies designed to evaluate the interrelationships amongst annoyance and wind turbine noise, as well as the influence of subjective variables such as attitude and noise sensitivity, Pedersen and Persson Waye [13-15] showed that people tend to notice sound from wind turbines almost linearly with increasing sound pressure level. Briefly, Pedersen and Persson Waye conducted cross-sectional studies (in 2004: n = 351; in 2007: n = 754) and gave people questionnaires regarding housing and satisfaction with the living environment, including questions about degree of annoyance experienced outdoors and indoors and sensitivity to environmental factors, wind turbines (noise, shadows, and disturbances), respondents' level of perception and annoyance, and verbal descriptors of sound and perceptual characteristics. The third section had questions about chronic health (e.g., diabetes, tinnitus, cardiovascular diseases), general wellbeing (e.g., headache, undue tiredness feeling tensed/stressed, irritable) and normal sleep habits (e.g., quality of sleep, whether or not sleep was disturbed by any noise source). The last section comprised questions on employment and working hours. Of import, the purpose of the study was masked in the questionnaires, which was done to reduce the potential for survey bias.

Of the 754 respondents involved in the Pedersen and Persson Waye study [14], 307 (39%) noticed sound from wind turbines outside their dwelling (range of sound pressure level: < 32.5, 32.5-35.0, 35.0-37.5, 37.5-40.0, and > 40.0 dB(A)) and the proportion of respondents who noticed sound increased almost linearly with increasing noise. In the 37.5-40.0 dB(A) range, 76% of the 71 respondents reported that they noticed sound from the wind turbines; 90% of respondents (n = 18) in the > 40.0 dB(A) category noticed sound from the wind turbines. The odds of noticing sound increased by 30% for each increase in dB(A) category. When data from both studies [13,14] were combined (n = 1095) results were the same: the proportion of respondents who noticed sound from wind turbines showed increased almost linearly with increasing sound pressure level from roughly 5-15% of people noticing noise at 29 dB(A) to 45-90% noticing noise at 41 dB(A)[15].

In 2011 Pedersen [25] reported on the results of three cross-sectional studies conducted in two areas of Sweden (a flat rural landscape (n = 351) and suburban sites with hilly terrain (n = 754) and one location in the Netherlands (flat landscape but with different degrees of road traffic intensity (n = 725)) designed assess the relationship between wind turbine noise and possible adverse health effects. Questionnaires were mailed to people in the three areas to obtain information about annoyance and health effects in response to wind turbines noise. Pedersen included questions about several potential environmental stressors and did not allow participants to know that the focus of the study was on wind turbine noise, again in an attempt to reduce self-reporting survey bias. For each respondent, sound pressure levels (dB(A)) were calculated for nearby wind turbines. The questionnaires were designed to obtain information about people's response to noise (i.e., annoyance), diseases or symptoms of impaired health (i.e., chronic disease, diabetes, high blood pressure, cardiovascular disease, tinnitus, impaired hearing), stress symptoms (i.e., headache, undue tiredness, feeling tense or stressed, feeling irritable), and disturbed sleep (i.e., interruption of the sleep by any noise source). Results showed that the frequency of those annoyed with wind turbines was related to an increase in sound pressure level as shown by odds ratios (OR) with 95% confidence intervals (CI) greater than 1.0. Sleep interruption was associated with sound level in two of the three studies (the areas with flat terrain), but unlike the finding that people tend to notice sound from wind turbines almost linearly with increasing sound pressure level, sleep disturbance did not increase gradually with noise levels, but spiked at 40 dBA and 45 dBA.

### 2. A proportion of people that notice sound from wind turbines find it annoying

Results of the Pedersen and Persson Waye studies [13-15] also suggested that the proportion of participants who were fairly annoyed or very annoyed remained quite level through the 29-37 dB(A) range (no more than roughly 5%) but increased at noise levels above 37 dB(A), with peaks at 38 db(A) and 41 dB(A), where up to 30% of people were very annoyed. Respondents in the cross-sectional studies (and other studies [12]) noted that swishing, whistling, resounding and pulsating/throbbing were the sound characteristics that were most highly correlated with annoyance by wind turbine noise among respondents who noticed the noise outside their dwellings. This was also found by Leventhall [16]. Seven percent of respondents (n = 25) from the Pedersen and Persson Waye study [13] were annoyed by noise from wind turbines indoors, and this was related to noise category; 23% (n = 80) were disturbed in their sleep by noise. Of the 128 respondents living at sound exposure above 35.0 dB(A), 16% (n = 20) stated that they were disturbed in their sleep by wind turbine noise. The authors comment that some people may find wind turbine noise more annoying than that of other types of noise (e.g., airplane and traffic) experienced at similar decibel levels.

Similar results were shown by Pedersen and Persson Waye [14]: a total of 31 of the 754 respondents said they were annoyed by wind turbine noise. In the < 32.5 to the 37.5 dB(A) category 3% to 4% of people said they were annoyed by wind turbine noise; in the 37.5-40.0 dB(A) category, 6% of the 71 respondents were annoyed; and in the > 40.0 category, 15% of 20 of respondents said they were annoyed by wind turbine noise. In addition, 36% of those 31 respondents who were annoyed by wind turbine noise reported that their sleep was disturbed by a noise source. Nine percent of those 733 respondents not annoyed said their sleep was disturbed by a noise source. Results of Pedersen [25] showed similar results: the frequency of those annoyed was related to an increase in sound pressure level. Moreover, self reported health effects like feeling tense, stressed, and irritable, were associated with noise annoyance and not to noise itself (OR and 95%CI > 1.0). Sleep interruption, however, was associated with sound level and annoyance (OR and 95%CI > 1.0). Pedersen notes that this finding is not necessarily evidence of a causal relationship between wind turbine noise and stress but may be explained by cognitive stress theory whereby "an individual appraises an environmental stressor, such as noise, as beneficial or not, and behaves accordingly". In other words, it appears that it is the change in the environment that is associated with the self-reported health effects, not the presence of wind turbines themselves.

Keith et al. [17] proposed that in a quiet rural setting, the predicted sound level from wind turbines should not exceed 45 dB(A) at a sensitive receptor location (e.g., residences, hospitals, schools), a value below the World Health Organization guideline for sleep and speech disturbance, moderate annoyance and hearing impairment. The authors [17] suggest this level of noise could be expected to result in a 6.5% increase in the percentage of highly annoyed people. Since publication of the Keith et al. study, the WHO Europe Region has released new Night Noise Guidelines for Europe [8] and state that: "The new limit is an annual average night exposure not exceeding 40 decibels (dB), corresponding to the sound from a quiet street in a residential area". The value of 40 dB is considered the lowest observed adverse effect level (LOAEL) for night noise based on the finding that an average night noise level over a year of 30-40 dB can result in a number of effects on sleep such as body movements, awakening, self-reported sleep disturbance and arousals [8]. The WHO states that even in the worst cases these effects seem modest [8].

### 3. Annoyance is not only related to wind turbine noise but also to subjective factors like attitude to visual impact, attitude to wind turbines and sensitivity to noise

Pedersen and Persson Waye [13] revealed that attitude to visual impact, attitude to wind turbines in general, and sensitivity to noise were also related to the way people perceived noise from turbines. For example, 13% of the variance in annoyance from wind farms could be explained by noise and the odds that respondents would be annoyed by noise from wind turbines increased 1.87 times from one sound category to the next. When noise and attitude to visual impact was statistically assessed, 46% of the variance in annoyance from wind farms could be explained and the odds that respondents would be annoyed from wind turbines increased 5.05 times from one sound category to the next. Statistical analyses showed that while attitude to wind turbines in general and sensitivity to noise were also related to annoyance, they did not have a greater influence on annoyance than visual effect. Building on their 2004 paper, Pedersen and Persson Waye [14] conducted a cross-sectional study in seven areas in Sweden across dissimilar terrains and with different degrees of urbanization. Three areas were classified as suburban; four as rural. Noise annoyance related to wind turbines was also statistically related to whether or not people live in suburban or rural areas and landscape (flat vs. hilly/complex). Visual impact has come out as a stronger predictor of noise annoyance than wind turbine noise itself. People who economically benefit from wind turbines had significantly decreased levels of annoyance compared to individuals that received no economic benefit, despite exposure to similar sound levels [18].

One suggestion of the difference between rural and suburban areas is level of background sound and interestingly, perception and annoyance was associated with type of landscape, "indicating that the wind turbine noise interfered with personal expectations in a less urbanised area... pointing towards a personal factor related to the living environment" [14]. The authors also concluded that visual exposure enhances the negative associations with turbines when coupled with audible exposure. They also point out that this study showed that aesthetics play a role in annoyance: "respondents who think of wind turbines as ugly are more likely to appraise them as not belonging to the landscape and therefore feel annoyed" [14].

In 2007 Pedersen et al. [19] conducted a grounded theory study to gain a deeper understanding of how people living near wind turbines perceive and are affected by them. Findings indicated that the relationship between exposure and response is complex and possibly influenced by variables not yet identified, some of which are nonphysical. The notion that wind turbines are "intruders" is a finding not reported elsewhere. A conclusion of this paper is that when the impacts of wind turbines are assessed, values about the living environment are important to consider as values are firmly rooted within a personality and difficult to change.

In 2008, Pedersen and Larsman [20] conducted a study to assess visibility of wind turbines, visual attitude and vertical visual angle (VVA) in different landscapes. This study follows up on the findings of previous work showing a relationship between noise annoyance in people living near wind turbines and the impact of visual factors as well as an individual's attitude toward noise [13-15,25]. Overall, Pedersen and Larsman concluded that respondents in a landscape where wind turbines could be perceived as contrasting with their surroundings (i.e., flat areas) had a greater probability of noise annoyance than those in hilly areas (where turbines were not as obvious), regardless of sound pressure level, if they thought wind turbines were ugly, unnatural devices that would have a negative impact on the scenery. The enhanced negative response could be linked to aesthetical response, rather than to multi-modal effects of simultaneous auditory and visual stimulation. Moreover, VVA was associated with noise annoyance, especially for respondent who could see at least one wind turbine from their dwelling, if they were living in flat terrain and rural areas. Pedersen and Larsman suggest that these results underscore the importance of visual attitude towards the noise source when exploring response to environmental noise. In 2010 Pedersen et al. [21] hypothesized that if high levels of background sound can reduce annoyance by masking the noise from a wind farm, then turbines could cause less noise annoyance when placed next to motorways instead of quiet agricultural areas. In general, the hypothesis was not supported by the available data [15], further providing support for the notion of visual cue being a strong driver of annoyance.

### 4. Turbines are designed not to pose a risk of photo-induced epilepsy

Harding et al. [22] and Smedley et al. [23] investigated the relationship between photo-induced seizures (i.e., photosensitive epilepsy) and wind turbine blade flicker (also known as shadow flicker). This is an infrequent event, typically modelled to occur less than 30 hours a year from wind turbine projects we have reviewed and would be most common at dusk and dawn, when the sun is at the horizon. Both studies suggested that flicker from turbines that interrupt or reflect sunlight at frequencies greater than 3 Hz pose a potential risk of inducing photosensitive seizures in 1.7 people per 100,000 of the photosensitive population. For turbines with three blades, this translates to a maximum speed of rotation of 60 rpm. The normal practice for large wind farms is for frequencies well below this threshold.

Although shadow flicker from wind turbines is unlikely lead to a risk of photo-induced epilepsy there has been little if any study conducted on how it could heighten the annoyance factor of those living in proximity to turbines. It may however be included in the notion of visual cues. In Ontario it has been common practice to attempt to ensure no more than 30 hours of shadow flicker per annum at any one residence.

### 5. The human ear responds to infrasound

Infrasound is produced by physiological processes like respiration, heartbeat and coughing, as well as man-made sources like air conditioning systems, vehicles, some industrial processes and wind turbines. Salt and Hullar [24] provide data to suggest that the assumption that infrasound presented at an amplitude below what is audible has no influence on the ear is erroneous and summarize the results of previous studies that show a physiological response of the human ear to low frequency noise (LFN) and infrasound. At very low frequencies the outer hair cells (OHC) of the cochlea may be stimulated by sounds in the inaudible range. Salt and Hullar hypothesize that "if infrasound is affecting cells and structures at levels that cannot be heard this leads to the possibility that wind turbine noise could be influencing function or causing unfamiliar sensations". These authors do not test this hypothesis in their paper but suggest the need for further research.

To assess the possibility that the operation of wind turbines may create unacceptable levels of low frequency noise and infrasound, O'Neal et al. [26] conducted a study (commissioned by a wind energy developer, NextEra Energy Resources, LLC) to measure wind turbine noise outside and within nearby residences of turbines. At the Horse Hollow Wind Farm in Taylor and Nolan Counties, Texas, broadband (A-weighted) and one-third octave band data (3.15 hertz to 20,000 hertz bands) were simultaneously collected from General Electric (GE) 1.5sle (1.5 MW) and Siemens SWT-2.3-93 (2.3 MW) wind turbines. Data were collected outdoors and indoors over the course of one week under a variety of operational conditions (it should be noted that wind speeds were low during the measurements; between 3.2 and 4.1 m/s) at two distances from the nearest wind turbines: 305 meters and 457 meters. O'Neal et al. found that the measured low frequency sound and infrasound at both distances (from both turbine types at maximum noise conditions) were less than the standards and criteria published by the cited agencies (e.g., UK DEFRA (Department for Environment, Food, and Rural Affairs); ANSI (American National Standards Institute); Japan Ministry of Environment). The authors concluded that results of their study suggest that there should be no adverse public health effects from infrasound or low frequency noise at distances greater than 305 meters from the two wind turbine types measured.

### Popular Literature

Scientific studies peer reviewed and published in scientific journals are one way of disseminating information about wind turbines and health effects. The general public does not always have access to scientific journals and often get their information, and form opinions, from sources that are less accountable (e.g., the popular literature and internet). Some of the same key words used to obtain references from the primary literature were entered into the common internet search engine Google: "health effects wind farms" returned 300,000 hits; "health effects wind turbines" returned 120,000 hits; "annoyance wind turbines" returned 185,000 hits and "sleep disturbance wind turbines" returned 19,500 hits. What is apparent is that numerous websites have been constructed by individuals or groups to support or oppose the development of wind turbine projects, or media sites reporting on the debate. Often these websites state the perceived impacts on, or benefits to, human health to support the position of the individual or group hosting the website. The majority of information posted on these websites cannot be traced back to a scientific, peer-reviewed source and is typically anecdotal in nature. In some cases, the information contained on and propagated by internet websites and the media is not supported, or is even refuted, by scientific research. This serves to spread misconceptions about the potential impacts of wind energy on human health, which either fuels or diminishes opposition to wind turbine project development.

Works by Dr. Michael Nissenbaum conducted at Mars Hill and Vinalhaven Maine [27] and Dr. Nina Pierpont in New York [28] seem to be the primary popular literature studies referenced on websites. These works suggest a causal link between human health effects and wind turbines. Works by Dr. Robert McMurtry and Carmen Krogh, and Lorrie Gillis, Carmen Krogh and Dr. Nicholas Kouwen [29] have also been used to suggest a relationship between health and turbines. These works have been presented as reports or as slide presentations on websites and authors of these studies have presented their findings in various forua such as invited lectures, affidavits, public meetings and open houses. Briefly, Nissenbaum evaluated 22 exposed adults (defined as living within 3500 ft of an arrangement of 28 1.5 MW wind turbines) and 27 unexposed adults (living about 3 miles away from the nearest turbine). Participants were interviewed and asked a number of questions about their perceived health, levels of stress and reliance on prescription medications in relation to the turbines [27].

In 2009, a book entitled Wind Turbine Syndrome: A Report on a Natural Experiment by Dr. Nina Pierpont, was self-published and describes "Wind Turbine Syndrome", the clinical name Dr. Pierpont coined for the collection of symptoms reported to her by people residing near wind turbines [28]. The book describes a case series study she conducted involving interviews of 10 families experiencing adverse health effects and who reside near wind turbines. Similar to the process followed by Nissenbaum, people living in proximity wind turbines were interviewed about their health. For all of these works, self-reported symptoms generally included sleep disturbance, headache, tinnitus (ringing in the ears), ear pressure, dizziness, vertigo, nausea, visual blurring, tachycardia (rapid heart rate), irritability, problems with concentration and memory and panic episodes. These symptoms have been purported to be associated with proximity to wind turbines, and specifically, to the infrasound emitted by the turbines. It should be noted that of the 351 people assessed by Pedersen and Persson Waye [13], 26% (91) reported chronic health issues (e.g., diabetes, tinnitus, cardiovascular diseases), but these issues were not statistically associated with noise levels. Results of Pedersen [25] showed similar results: self reported health effects like feeling tense, stressed, and irritable, were associated with noise annoyance and not to noise itself. Sleep interruption, however, was associated with sound level and annoyance.

In 2007, Alves-Pereira and Castelo Branco http://www.wind-watch.org/documents/industrial-wind-turbines-infrasound-and-vibro-acoustic-disease-vad/ issued a press-release suggesting that their research demonstrated that living in proximity to wind turbines has led to the development of vibro-acoustic disease (VAD) in nearby home-dwellers. It appears that this research has only been presented at a conference, has not been published in a peer-reviewed journal nor has it undergone thorough scientific review. Moreover, Alves-Pereira and Castelo Branco appear to be the primary researchers that have promulgated VAD as a hypothesis for adverse health effects and wind turbines. Indeed, Dr. Pierpont has noted that VAD is not the same "wind turbine syndrome" [28].

To date, these studies have not been subjected to rigorous scientific peer review, and given the venue for their distribution and limited availability of data, it is extremely difficult to assess whether or not the information provided is reliable or valid. What is apparent, however, is that these studies are not necessarily scientifically defensible: they do not contain noise measurements, only measured distances from study participants to the closest turbines; they do not have adequate statistical representation of potential health effects; only limited rationale is provided for the selection of study participants (in some cases people living in proximity to turbines have been excluded from the study); they suffer from a small number of participants and appear to lack of objectivity as authors are also known advocates who oppose wind turbine developments. Unlike the questionnaires used by Pedersen et al. [13-15,25], the purpose of the studies are not hidden from participants. In fact, the selection process is highly biased towards finding a population who believes they have been affected by turbines. This is not an attempt to discount the self-reported health issues of residents living near wind turbines. Rather, it points out that the self-reported health issues have not been definitively linked to wind turbines.

What the peer reviewed literature and popular literature have in common is the conclusion that wind turbines can be a source of annoyance for some people. Of note are the different reasons and possible causes for annoyance. In the peer reviewed studies, annoyance tends to peak in the > 35 dB(A) range but tends to be more strongly related to subjective factors like visual impact, attitude to wind turbines in general (benign vs. intruders) and sensitivity to noise rather than noise itself from turbines. In the popular literature, health outcomes tend to be more strongly related to distance from turbines and the claim that infrasound is the causative factor. Though sound pressure level in most of the peer reviewed studies was scaled to dB(A) (but refer to O'Neal et al. [26] for actual measurements of low frequency noise and infrasound), infrasound is a component of the sound measurements and was inherently accounted for in the studies.

### Annoyance

Studies on the health effects of wind turbines, both published and peer-reviewed and presented in the popular literature, tend to conclude that wind turbines can cause annoyance for some people. A number of governmental health agencies agree that while noise from wind turbines is not loud enough to cause hearing impairment and are not causally related to adverse effects, wind turbines can be a source of annoyance for some people [1,30-34].

It has been hypothesized that the self reported health effects (e.g., sleep disturbance, headache, tinnitus (ringing in the ears), ear pressure, dizziness, vertigo, nausea, visual blurring, tachycardia (rapid heart rate), irritability, problems with concentration and memory, and panic episodes) are related to infrasound emitted from wind turbines [28]. Studies where biological effects were observed due to infrasound exposure were conducted at sound pressure levels (e.g., 145 dB and 165 dB [5,16]; 130 dB [7]) much greater than what is produced by wind turbines (e.g., see O'Neal et al. [26]). Infrasound is not unique to wind turbines but is ubiquitous in the environment due to natural and man-made sources, meaning that people living near wind turbines were exposed to infrasound prior to turbine operation. For example, Berglund and Hassmen [35] reported that infrasound (a component of low frequency sound) is emitted from road vehicles, aircraft, industrial machinery, artillery and mining explosions, air movement machinery including wind turbines, compressors, and air-conditioning units, and Leventhall [5] reported that infrasound comes from natural sources like meteors, volcanic eruptions and ocean waves. Indeed, many mammals communicate using infrasound [36]. Given the low sound pressure levels of infrasound emitted from wind turbines and the ubiquitous nature of these sounds, the hypothesis that infrasound is a causative agent in health effects does not appear to be supported.

Peer reviewed and scientifically defensible studies suggest that annoyance and health effects are more strongly related to subjective factors like visual impact and attitude to wind turbines rather than to noise itself (both audible and inaudible [i.e., infrasound]). Indeed, many of the self reported health effects are associated with numerous issues, many of which can be attributed to anxiety and annoyance (e.g., Pedersen 2011 [25]). Shargorodsky et al. [37] published that roughly 50 million adults in the United States reported having tinnitus, which is statistically correlated (based on 14,178 participants) to age, racial/ethnic group, hypertension, history of smoking, loud leisure-time, firearm, and occupational noise, hearing impairment and generalized anxiety disorder (based on 2265 participants) identified using a World Health Organization Composite Diagnostic Interview). In fact, the odds of tinnitus being related to anxiety disorder were greatest for any of the variables tested. Folmer and Griest [38], based on a study of 174 patients undergoing treatment for tinnitus at the Oregon Health Sciences University Tinnitus Clinic between 1994 and 1997, reported that insomnia is associated with greater severity of tinnitus. Insomnia is also associated with anxiety and annoyance. Bowling et al. [39] described statistically that people's perceptions of neighbourhood environment can influence health. Perceptions of problems in the area (e.g., noise, crime, air quality, rubbish/litter, traffic, graffiti) were predictive of poorer health score. In their 2003 publication Henningsen and Priebe [40] discussed the characteristics of "New Environmental Illness", illnesses where patients strongly believe their symptoms are caused by environmental factors, even though symptoms are not consistent with empirical evidence and medically unexplained. A key component to such illnesses is the patient's attitude toward the source of the environmental factor. What is more, health effects from annoyance have been shown to be mitigated though behavioural and cognitive behavioural interventions [30,41], lending support to Pedersen's [25] conclusion that health effects can be explained by cognitive stress theory. In other words, it appears that it is the change in the environment that is associated with health effects, not a turbine-specific variable like infrasound.

## Conclusions

Wind power has been harnessed as a source of power around the world. Debate is ongoing with respect to the relationship between reported health effects and wind turbines, specifically in terms of audible and inaudible noise. As a result, minimum setback distances have been established world-wide to reduce or avoid potential effects for people living in proximity to wind turbines. People interested in this debate turn to two sources of information to make informed decisions: scientific peer-reviewed studies published in scientific journals and the popular literature and internet.

We found that conclusions of the peer reviewed literature differ in some ways from the conclusions of the studies published in the popular literature. What both types of studies have in common is the conclusion that wind turbines can be a source of annoyance for some people. In the peer reviewed studies, wind turbine annoyance and some reported health effects (e.g., sleep disturbance) have been statistically associated with wind turbine noise especially when found at sound pressure levels greater than 40 db(A), but found to be more strongly related to subjective factors like visual impact, attitude to wind turbines in general and sensitivity to noise. To date, no peer reviewed scientific journal articles demonstrate a causal link between people living in proximity to modern wind turbines, the noise (audible, low frequency noise, or infrasound) they emit and resulting physiological health effects. In the popular literature, self-reported health outcomes and annoyance are related to distance from turbines and the claim is made that infrasound is the causative factor for the reported effects, even though sound pressure levels are not measured. Infrasound is not unique to wind turbines and the self reported health effects of people living in proximity to wind turbines are not unique to wind turbines. Given that annoyance appears to be more strongly related to visual cues and attitude than to noise itself, self reported health effects of people living near wind turbines are more likely attributed to physical manifestation from an annoyed state than from infrasound. This hypothesis is supported by the peer-reviewed literature pertaining to environmental stressors and health.

The authors have spent countless hours at community public consultation events hosted by proponents announcing new projects and during updates to their environmental assessment process. Historically, citizens' concerns about wind turbine projects appeared to involve potential impact on property values and issues surrounding avian and bat mortality. Increasingly in North America the issue surrounding fears of potential harm to residents' health have come to the forefront of these meetings. It is clear that the announcement of a new project can led to a heightened sense of anxiety and annoyance in some members of the public, even prior to construction and operation of a wind turbine project. The authors have been involved in all manner of risk communication, consultation and risk assessment projects in the energy sector in Canada and it has been our experience that this heightened sense of annoyance, agitation or fear is not unique to the wind turbine sector. Whether the proposed project is a wind turbine, gas-fired station, coal plant, nuclear power plant, or energy-from-waste incinerator we have seen a level of concern in a sub-set of the population that goes well beyond anything that would be considered the traditional sense of not-in-my-back-yard (NIMBY). These people genuinely are fearful about the potential health effects that the project may cause, regardless of the outcomes of quantitative assessments that demonstrate that there is a *de minimus *of potential risk in living next to a particular facility. The literature and our own experience highlight the need for informative discussions between wind power developers and community members in order to attempt to reduce the level of apprehension. We encourage continued dialogue between concerned citizens and developers once projects become operational.

Canadian public health agencies subscribe to the World Health Organization definition of health. *"Health is a state of complete physical, mental and social well-being and not merely the absence of infirmity or disease"*, a quote often used by both sides of the wind turbine debate. We believe that the primary role of the environmental health/risk assessment practitioner is to ensure that physiological manifestation of infirmity or disease is not predicted to occur from exposure to an environmental contaminant. In terms of wind power, ethics dictate an honest reporting of the issues surrounding annoyance and the fact that it appears that a limited number of people have self-reported health effects that may be attributed to the indirect effects of visual and attitudinal cue. We believe that any physiological based effect can be mitigated through the use of appropriate setback distances. However, it is not clear that for this hypersensitive annoyed population that any set back distance could mitigate the indirect effects. Therefore, it is up to our elected officials and ministerial staff when establishing an energy source hierarchy to weigh all of the information before them to determine the trade-offs between "mental and social well-being" of these individuals against the larger demand for energy and its source.

A number of governmental health agencies agree that while noise from wind turbines is not loud enough to cause hearing impairment and are not causally related to adverse effects, wind turbines can be a source of annoyance for some people. Ultimately it is up to governments to decide the level of acceptable annoyance in a population that justifies the use of wind power as an alternative energy source.

Assessing the effects of wind turbines on human health is an emerging field, as demonstrated by the limited number of peer-reviewed articles published since 2003. Conducting further research into the effects of wind turbines (and environmental change) on human health, emotional and physical, as well as the effect of public consultation with community groups in reducing pre-construction anxiety, is warranted. Such an undertaking should be initiated prior to public announcement of a project, and could involve baseline community health and attitude surveys, baseline noise and infrasound monitoring, observation and questionnaires administered to public during the siting and assessment process, noise modeling and then post-construction follow-up on all of the aforementioned aspects. Regardless it would be imperative to ensure robust study design and a clear statement of purpose prior to study initiation.

We believe that research of this nature should be undertaken by multi-disciplinary teams involving, for example, acoustical engineers, health scientists, epidemiologists, social scientists and public health physicians. Ideally developers, government agencies, consulting professionals and non-government organizations would form collaborations in attempt to address these issues.

## List of Abbreviations

ANSI: American National Standards Institute; CI: Confidence intervals; dB(A): A-weighted decibels; DEFRA: Department for Environment, Food, and Rural Affairs; LFN: low frequency noise; LOAEL: lowest observed adverse effect level; MW: mega watt; O.Reg.: Ontario Regulation; OR: odds ratio; OHC: outer hair cells; REA: Renewable Energy Approval; SCI: Science Citation Index; SSCI: Social Sciences Citation Index; VAD: vibro-acoustic disease; VVA: vertical visual angle; WHO: World Health Organization

## Competing interests

In terms of competing interests (financial and non-financial), the authors work for a consulting firm and have worked with wind power companies. The authors are actively working in the field of wind turbines and human health. Dr. Ollson has acted as an expert witness for wind power companies during a number of legal hearings. Although we make this disclosure, we wish to reiterate that as independent scientific professionals our views and research are not influenced by these contractual obligations. The authors are environmental health scientists, trained and schooled, in the evaluation of potential risks and health effects of people and the ecosystem through their exposure to environmental issues such as wind turbines.

## Authors' contributions

LDK and CAO both researched and wrote the manuscript. Both authors read and approved the final version.
